# Supplementary material for: Pathological, Morphological, Cytogenomic, Biochemical and Molecular Data Support the Distinction between Colletotrichum cigarro comb. et stat. nov. and Colletotrichum kahawae
Source: Plants (Basel). 2020 Apr 14;9(4):502. doi: 10.3390/plants9040502 (PMC7238176; doi:10.3390/plants9040502)
Supplement: Supplementary file 1 [file plants-09-00502-s001.zip › Supplementary Figure 1.docx]

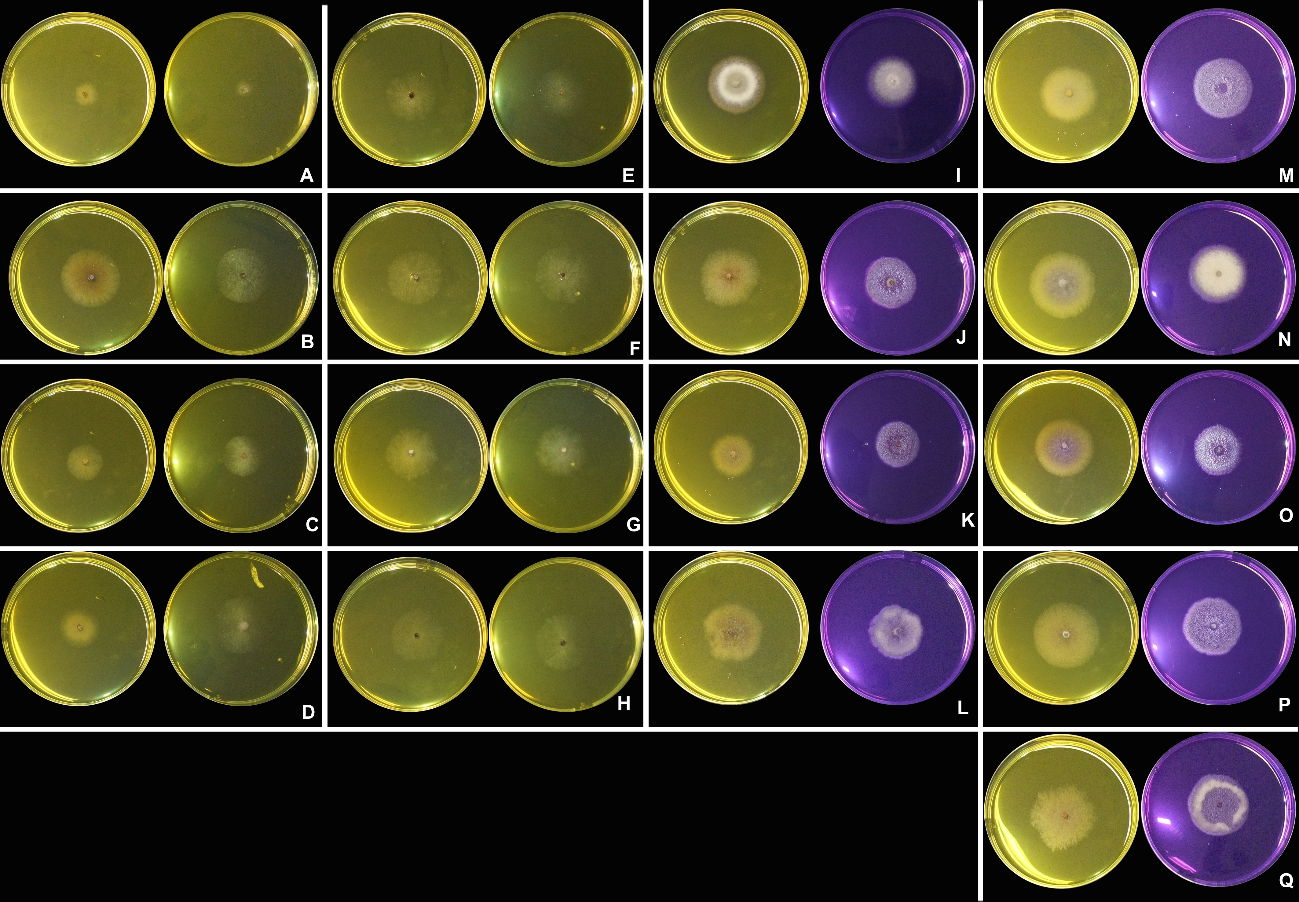


Supplementary Figure 1 – *Colletotrichum* species grown on 90 mm plates contained citric acid (left side of each letter) or ammonium tartrate (right side of each letter) as sole carbon sources, incubated at 25 °C and medium colour registered after 7 days as purple (substrate used) or yellow (substrate not used). CBD-causing isolates (*C. kahawae*): Ang29 (A), Ang67 (B), Bur2 (C), Cam1 (D), Cam5 (E), Mal2 (F), Que2 (G), Uga7 (H); non-CBD causing isolates (*C. cigarro*) ICMP 18539 (I), ICMP 18534 (J), CBS 237.49 (K), ICMP 12953 (L), PR432 (M), PR428 (N); *C. camelliae* ICMP 18542 (O); *C. aotearoa* ICMP 18537 (P); *C. gloesporioides* PR220 (Q).
