# Supplementary material for: Pathological, Morphological, Cytogenomic, Biochemical and Molecular Data Support the Distinction between Colletotrichum cigarro comb. et stat. nov. and Colletotrichum kahawae
Source: Plants (Basel). 2020 Apr 14;9(4):502. doi: 10.3390/plants9040502 (PMC7238176; doi:10.3390/plants9040502)
Supplement: Supplementary file 1 [file plants-09-00502-s001.zip › Supplementary Figure 2.docx]

CBS 237.49(*) *Hypericum* Germany

ICMP 18534 *Kunzea* New Zealand

100/1

ICMP 18542 *Camellia* USA

Uga7 *Coffea* Uganda

PR432 *Mangifera* Portugal

Mal2 *Coffea* Malawi

**ICMP 18539 *Olea* Australia**

ICMP 12953 *Persea* New Zealand

Cam5 *Coffea* Cameroon

Cam1 *Coffea* Cameroon

Ang29 *Coffea* Angola

PR428 *Camellia* Portugal

Ang67 *Coffea* Angola

Que2 *Coffea* Kenya

Bur2 *Coffea* Burundi

99/1

PR220 *Olea* Portugal

**ICMP 18537 *Coprosma* New Zealand**

0.1

*C. gloeosporioides*

*C. aotearoa*

*C. kahawae*

*C. cigarro*

*C. cigarro*

*C. camelliae*

ApMAT

Que2 *Coffea* Kenya

Bur2 *Coffea* Burundi

Mal2 *Coffea* Malawi

89/1

Ang67 *Coffea* Angola

Ang29 *Coffea* Angola

89/1

Cam5 *Coffea* Cameroon

Cam1 *Coffea* Cameroon

Uga7 *Coffea* Uganda

87/0.99

ICMP 18534 *Kunzea* New Zealand

CBS 237.49(*) *Hypericum* Germany

84/0.97

ICMP 12953 *Persea* New Zealand

PR428 *Camellia* Portugal

94/0.99

PR432 *Mangifera* Portugal

**ICMP 18539 *Olea* Australia**

88/1

ICMP 18542 *Camellia* USA

86/1

PR220 *Olea* Portugal

**ICMP 18537 *Coprosma* New Zealand**

0.1

*C. kahawae*

*C. camelliae*

*C. cigarro*

*C. aotearoa*

*C. gloeosporioides*

*apn25L*

Bur2 *Coffea* Burundi

Uga7 *Coffea* Uganda

Que2 *Coffea* Kenya

Mal2 *Coffea* Malawi

Cam5 *Coffea* Cameroon

Cam1 *Coffea* Cameroon

100/0.96

Ang29 *Coffea* Angola

Ang67 *Coffea* Angola

100/1

ICMP 18534 *Kunzea* New Zealand

CBS 237.49(*) *Hypericum* Germany

100/1

**ICMP 18539 *Olea* Australia**

ICMP 18542 *Camellia* USA

ICMP 12953 *Persea* New Zealand

PR428 *Camellia* Portugal

PR432 *Mangifera* Portugal

96/1

PR220 *Olea* Portugal

**ICMP 18537 *Coprosma* New Zealand**

0.01

*C. aotearoa*

*C. gloeosporioides*

*C. kahawae*

*C. camelliae*

*C. cigarro*

*C. cigarro*

*mat1-2-1*

Ang29 *Coffea* Angola

Uga7 *Coffea* Uganda

Que2 *Coffea* Kenya

Mal2 *Coffea* Malawi

Cam5 *Coffea* Cameroon

Cam1 *Coffea* Cameroon

Bur2 *Coffea* Burundi

Ang67 *Coffea* Angola

100/1

**ICMP 18539 *Olea* Australia**

CBS 237.49(*) *Hypericum* Germany

100/1

ICMP 18534 *Kunzea* New Zealand

99/1

PR428 *Camellia* Portugal

ICMP 18542 *Camellia* USA

43/0.72

PR432 *Mangifera* Portugal

ICMP 12953 *Persea* New Zealand

99/0.98

90/1

PR220 *Olea* Portugal

**ICMP 18537 *Coprosma* New Zealand**

0.01

*C. kahawae*

*C. camelliae*

*C. cigarro*

*C. cigarro*

*C. aotearoa*

*C. gloeosporioides*

*gs*

0.01

Ang29 *Coffea* Angola

Uga7 *Coffea* Uganda

Que2 *Coffea* Kenya

Mal2 *Coffea* Malawi

Cam5 *Coffea* Cameroon

Cam1 *Coffea* Cameroon

Bur2 *Coffea* Burundi

Ang67 *Coffea* Angola

**ICMP 18539 *Olea* Australia**

ICMP 18534 *Kunzea* New Zealand

CBS 237.49(*) *Hypericum* Germany

PR428 *Camellia* Portugal

ICMP 12953 *Persea* New Zealand

ICMP 18542 *Camellia* USA

PR432 *Mangifera* Portugal

**ICMP 18537 *Coprosma* New Zealand**

PR220 *Olea* Portugal

100/1

60/0.96

81/1

60/1

100/1

57/1

77/1

9/0.97

*C. kahawae*

*C. camelliae*

*C. cigarro*

*C. cigarro*

*C. aotearoa*

*C. gloeosporioides*

*cas1*

PR220 Olea Portugal

PR428 *Camellia* Portugal

**ICMP 18537** ***Coprosma* New Zealand**

Ang29 *Coffea* Angola

Ang67 *Coffea* Angola

Bur2 *Coffea* Burundi

Que2 *Coffea* Kenya

Mal2 *Coffea* Malawi

Uga7 *Coffea* Uganda

96/0.99

Cam1 *Coffea* Cameroon

Cam5 *Coffea* Cameroon

100/1

ICMP 18534 *Kunzea* New Zealand

**ICMP 18539** ***Olea* Australia**

83/0.68

CBS 237.49(*) *Hypericum* Germany

98/0.74

96/0.92

ICMP 18542 *Camellia* USA

98/1

PR432 *Mangifera* Portugal

97/1

ICMP 12953 *Persea* New Zealand

98/0.94

91/0.97

0.1

*C. kahawae*

*C. camelliae*

*C. cigarro*

*C. cigarro*

*C. aotearoa*

*C. gloeosporioides*

*vosA*

PR220 *Olea* Portugal

**ICMP 18537** ***Coprosma* New Zealand**

Ang29 *Coffea* Angola

Ang67 *Coffea* Angola

Cam5 *Coffea* Cameroon

Cam1 *Coffea* Cameroon

Mal2 *Coffea* Malawi

Uga7 *Coffea* Uganda

Bur2 *Coffea* Burundi

Que2 *Coffea* Kenya

84/0.98

97/1

ICMP 18534 *Kunzea* New Zealand

CBS 237.49(*) *Hypericum* Germany

82/1

66/1

ICMP 12953 *Persea* New Zealand

PR428 *Camellia* Portugal

100/1

**ICMP 18539** ***Olea* Australia**

PR432 *Mangifera* Portugal

97/0.92

95/1

ICMP 18542 *Camellia* USA

42/0.94

18/1

0.1

*C. kahawae*

*C. camelliae*

*C. cigarro*

*C. aotearoa*

*C. gloeosporioides*

*siRNA*

Ang29 *Coffea* Angola

Uga7 *Coffea* Uganda

Que2 *Coffea* Kenya

Mal2 *Coffea* Malawi

Cam5 *Coffea* Cameroon

Cam1 *Coffea* Cameroon

Bur2 *Coffea* Burundi

Ang67 *Coffea* Angola

ICMP 18534 *Kunzea* New Zealand

CBS 237.49(*) *Hypericum* Germany

100/1

ICMP 12953 *Persea* New Zealand

**ICMP 18539 *Olea* Australia**

PR428 *Camellia* Portugal

PR432 *Mangifera* Portugal

ICMP 18542 *Camellia* USA

PR220 *Olea* Portugal

**ICMP 18537 *Coprosma* New Zealand**

0.01

100/1

100/1

100/1

100/1

100/1

100/1

*C. kahawae*

*C. camelliae*

*C. cigarro*

*C. aotearoa*

*C. gloeosporioides*

*cellwall*

Bur2 *Coffea* Burundi

Uga7 *Coffea* Uganda

Que2 *Coffea* Kenya

Mal2 *Coffea* Malawi

88/1

CBS 237.49(*) *Hypericum* Germany

**ICMP 18539 *Olea* Australia**

ICMP 18534 *Kunzea* New Zealand

ICMP 12953 *Persea* New Zealand

Cam5 *Coffea* Cameroon

Cam1 *Coffea* Cameroon

PR428 *Camellia* Portugal

Ang67 *Coffea* Angola

Ang29 *Coffea* Angola

PR432 *Mangifera* Portugal

82/0.87

ICMP 18542 *Camellia* USA

89/1

PR220 *Olea* Portugal

**ICMP 18537 *Coprosma* New Zealand**

0.01

*C. kahawae*

*C. camelliae*

*C. cigarro*

*C. aotearoa*

*C. gloeosporioides*

*C. kahawae*

*tub2*

**Supplementary Figure 2** - Fifty percent majority rule consensus tree from a Bayesian analysis based single loci *apn25L* (part of Apn2 gene), ApMAT (intergenic spacer between the 3’ end of the apn2 gene and the mating type gene mat1-2-1), *mat1-2-1* (mating-type gene), *gs* (intron 2 of the glutamine synthetase gene), *cas1* (appressorium-specific protein), *cellwall* (cell wall protein), *siRNA* (a putative argonaute siRNA chaperone complex subunit), *vosA* (developmental regulator), and *tub2* (beta-tubulin) for isolates under study. The RAxML bootstrap support values (BS) and Bayesian posterior probability (PP) are displayed at the nodes (BS/PP). The tree was rooted to *Colletotrichum gloesporioides* (PR220). The scale bar indicates 0.1 or 0.01 expected changes per site. Ex-type cultures are emphasised in **bold**. (*) ex-type or authentic culture of synonymized taxon.
