# Supplementary material for: Pathological, Morphological, Cytogenomic, Biochemical and Molecular Data Support the Distinction between Colletotrichum cigarro comb. et stat. nov. and Colletotrichum kahawae
Source: Plants (Basel). 2020 Apr 14;9(4):502. doi: 10.3390/plants9040502 (PMC7238176; doi:10.3390/plants9040502)
Supplement: Supplementary file 1 [file plants-09-00502-s001.zip › Supplementary Figure 3.docx]

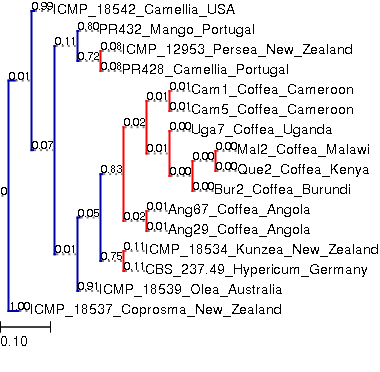


**Supplementary Figure 3 -** Maximum likelihood solution generated by bPTP (a Bayesian implementation of the Poisson tree process model) based on the nine-locus concatenated tree obtained in RaXML. The numbers in each node represents support value. The red color indicates the PTP suggested species.

http://species.h-its.org/ptp/result/?job_id=39620&email=anacgpcabral@gmail.com

# Most supported partition found by simple heuristic search

Species 1 (support = 1.000)

ICMP_18537_Coprosma_New_Zealand

Species 2 (support = 0.986)

ICMP_18542_Camellia_USA

Species 3 (support = 0.906)

ICMP_18539_Olea_Australia

Species 4 (support = 0.803)

PR432_Mango_Portugal

Species 5 (support = 0.723)

ICMP_12953_Persea_New_Zealand,PR428_Camellia_Portugal

Species 6 (support = 0.833)

Cam1_Coffea_Cameroon,Cam5_Coffea_Cameroon,Uga7_Coffea_Uganda,Mal2_Coffea_Malawi,Que2_Coffea_Kenya,Bur2_Coffea_Burundi,Ang67_Coffea_Angola,Ang29_Coffea_Angola

Species 7 (support = 0.753)

ICMP_18534_Kunzea_New_Zealand,CBS_237.49_Hypericum_Germany
