# Supplementary material for: Pathological, Morphological, Cytogenomic, Biochemical and Molecular Data Support the Distinction between Colletotrichum cigarro comb. et stat. nov. and Colletotrichum kahawae
Source: Plants (Basel). 2020 Apr 14;9(4):502. doi: 10.3390/plants9040502 (PMC7238176; doi:10.3390/plants9040502)
Supplement: Supplementary file 1 [file plants-09-00502-s001.zip › Supplementary Figure 4.docx]

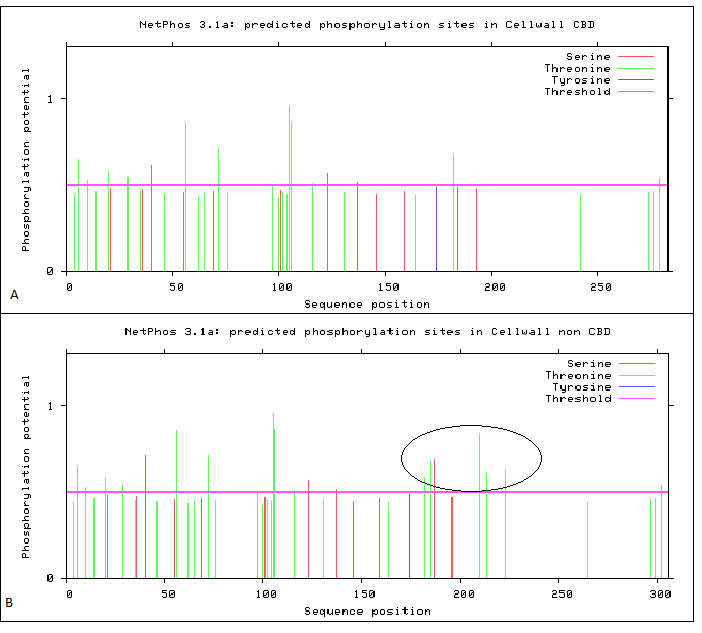


Supplementary Figure 4 – Graph illustrating the predictions of potential phosphorylation sites of cell wall protein of CBD-causing isolates (A) and non-CBD causing isolates (B). The black circle highlights major differences predicted. Higher phosphorylation potential indicates higher confidence in the prediction.
