# Supplementary material for: Pathological, Morphological, Cytogenomic, Biochemical and Molecular Data Support the Distinction between Colletotrichum cigarro comb. et stat. nov. and Colletotrichum kahawae
Source: Plants (Basel). 2020 Apr 14;9(4):502. doi: 10.3390/plants9040502 (PMC7238176; doi:10.3390/plants9040502)
Supplement: Supplementary file 1 [file plants-09-00502-s001.zip › Supplementary Figure 5.docx]

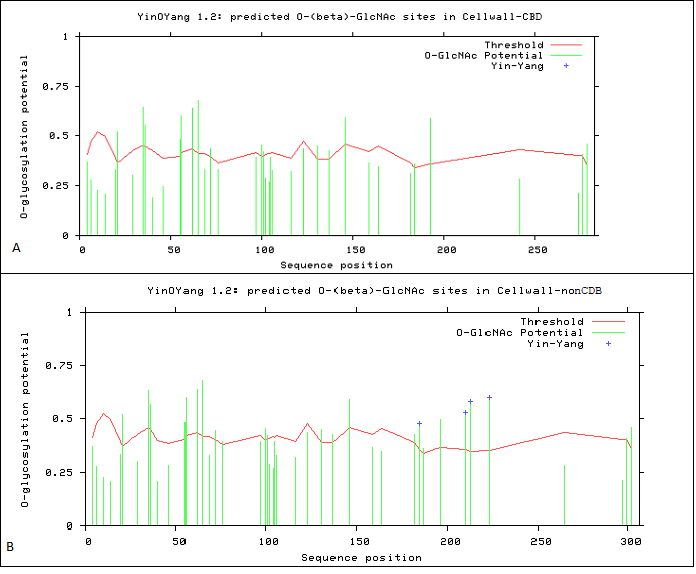


Supplementary Figure 5 - O-GlcNAc and NetPhos predictions across the length of the sequences of cell wall protein of CBD-causing isolates (A) and non-CBD causing isolates (B). The x-axis represents the sequence from N-terminal to C-terminal. Vertical lines (green) are O-GlcNAc potentials, more stringent surface measures are above the threshold (blue wavy horizontal) line. Small blue + on the green lines indicate YinYang sites predictions that correspond to Ser/Thr residues which are predicted to be O-GlcNAcylated as well as phosphorylated. Such sites may be reversibly and dynamically modified by O-GlcNAc or phosphate groups at different times in the cell [39].
